# Selective radical depolymerization of cellulose to glucose induced by high frequency ultrasound†

**DOI:** 10.1039/d0sc00020e

**Published:** 2020-02-06

**Authors:** Somia Haouache, Ayman Karam, Tony Chave, Jonathan Clarhaut, Prince Nana Amaniampong, José M. Garcia Fernandez, Karine De Oliveira Vigier, Isabelle Capron, François Jérôme

**Affiliations:** Institut de Chimie des Milieux et Matériaux de Poitiers, Université de Poitiers-CNRS 1 Rue Marcel Doré 86073 Poitiers France francois.jerome@univ-poitiers.fr; ICSM, Univ Montpellier, CNRS, CEA, ENSCM Bagnols-sur-Cèze France; Institute for Chemical Research, CSIC and University of Sevilla Americo Vespucio 49, Isla de la Cartuja 41092 Sevilla Spain; INRA, Site de la Géraudière 44316 Nantes France

## Abstract

The depolymerization of cellulose to glucose is a challenging reaction and often constitutes a scientific obstacle in the synthesis of downstream bio-based products. Here, we show that cellulose can be selectively depolymerized to glucose by ultrasonic irradiation in water at a high frequency (525 kHz). The concept of this work is based on the generation of H˙ and ˙OH radicals, formed by homolytic dissociation of water inside the cavitation bubbles, which induce the cleavage of the glycosidic bonds. The transfer of radicals on the cellulose particle surfaces prevents the side degradation of released glucose into the bulk solution, allowing maintaining the selectivity to glucose close to 100%. This work is distinguished from previous technologies in that (i) no catalyst is needed, (ii) no external source of heating is required, and (iii) the complete depolymerization of cellulose is achieved in a selective fashion. The addition of specific radical scavengers coupled to different gaseous atmospheres and ˙OH radical dosimetry experiments suggested that H˙ radicals are more likely to be responsible for the depolymerisation of cellulose.

## Abstract 2

Ultrasonic irradiation of cellulose at a high frequency induces its selective depolymerization to glucose at room temperature and atmospheric pressure within only a few minutes.

## Graphical abstract

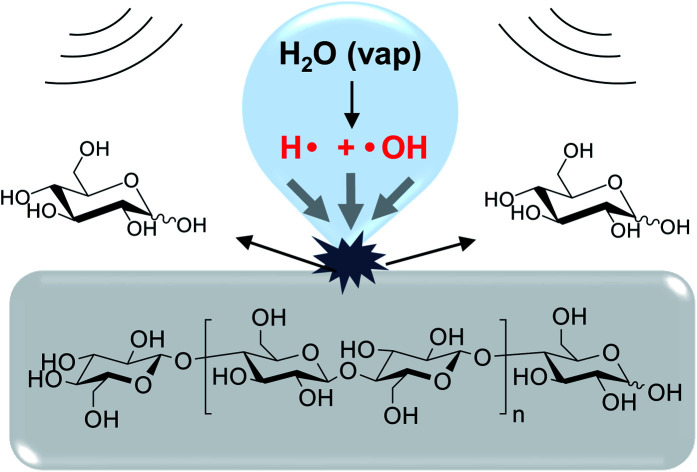

## Introduction

The depolymerisation of cellulose^1^ to glucose has become an important reaction paving the way to various biobased chemicals such as ethanol, furandicarboxylic acid, caprolactam, sorbitol, levulinic acid, γ-valerolactone, among many others.^2^ The hydrolysis of cellulose to glucose is however difficult to achieve and often constitutes an obstacle in the synthesis of downstream products.^3^ Indeed, this reaction requires overcoming high energy barriers of about 30–40 kcal mol^−1^,^3*a*,4^ essentially because cellulose exhibits a highly cohesive hydrogen bond network,^1,5^ strong van der Waals interactions,^6^ and electronic effects.^7^ To date, the selective hydrolysis of cellulose to glucose is performed by enzymatic routes.^8^ Although this route is deployed on a large scale for the production of ethanol, the price of enzymes as well as low space time yield and costly downstream purification processes hamper the large development of this route in the chemical industry. Alternatively, acid catalysts can also depolymerize cellulose, but they require harsh conditions and thus unfortunately afford glucose in low yield due to the formation of tar-like materials.^2,3^

The exploration of alternative technologies capable of selectively depolymerizing cellulose to glucose with a high efficiency is still an open scientific question.^9^ Supercritical water has emerged as a promising route for releasing glucose from cellulose^10^ and this process was even scaled-up by Renmatix.^11^ Solvent free technologies, aiming at producing concentrated feed of glucose, were also explored and one may cite the depolymerisation of cellulose by mechanocatalysis^12^ or by non-thermal atmospheric plasma (NTAP).^13^ With these solvent-free technologies, depolymerisation and repolymerization reactions occur simultaneously and processable water soluble oligosaccharides are obtained instead of monomeric glucose.

Here we report an alternative technology based on the use of high frequency ultrasound (HFUS). The ultrasonic irradiation of cellulose at a high frequency leads to a complete depolymerisation of cellulose to glucose, without any catalyst. In addition, the depolymerisation of cellulose induced by HFUS does not need any external source of heating or pressure, the energy being brought by the implosion of cavitation bubbles.^14^

When applied within a liquid, ultrasonic irradiation induce the nucleation, growth and collapse of gas and vapour filled bubbles. In contrast to the very popular low frequency ultrasound (<80 kHz) which mostly induces physical effects (shock waves, micro-jets, turbulences, *etc.*), irradiation of water at high frequencies (>150 kHz) mainly leads to the *in situ* formation of H˙ and ˙OH radicals resulting from the dissociation of water molecule.^15^ Once these cavitation bubbles implode, *in situ* formed radicals can recombine or react with solutes inducing chemical effects. Inspired by our previous works on NTAP,^13^ we conceived that these *in situ* produced radicals should theoretically induce the cleavage of the glycosidic bonds of cellulose.^16^ Being able to selectively depolymerize cellulose with H˙ and ˙OH radicals without side degradation of released glucose into the bulk solution is a challenging scientific task, which is addressed in this study.

When solid particles are present in an ultrasonic reactor, they act as nuclei for the formation and growth of cavitation bubbles. Close to a surface, the implosion of cavitation bubbles is very asymmetric and generates high-speed jets of liquid towards the surface, a good mean to concentrate radicals on a particle.^17^ Applied to cellulose, this physical principle should be an efficient mean to control the reaction selectivity by concentrating radicals on the cellulose particle surfaces, thus preventing side reactions of released glucose into the bulk solution.

## Result and discussion

**Scheme 1:**
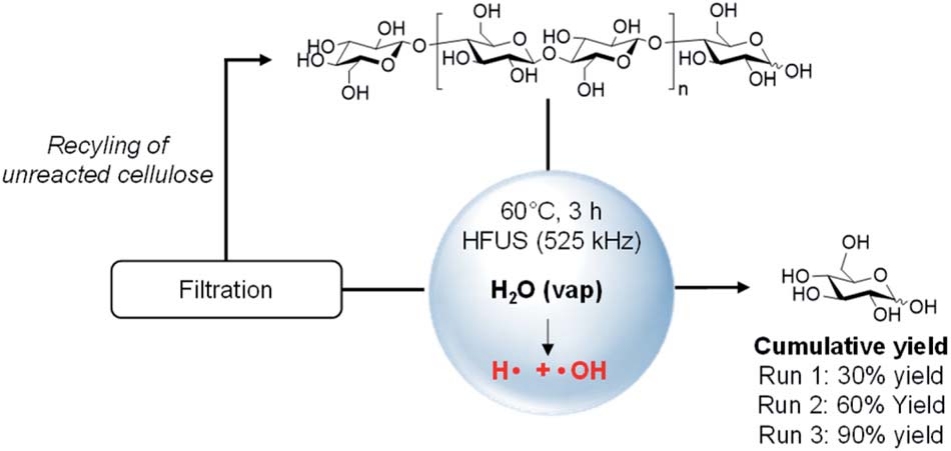
Depolymerisation of cellulose to glucose induced by HFUS.

To demonstrate the potential of the above concept, we first subjected microcrystalline cellulose (MCC, Avicel PH 200) to an ultrasonic irradiation at 525 kHz (acoustic power density of 0.36 W mL^−1^) in water, at 60 °C, and under atmospheric pressure of air. Analysis of the products formed was performed by HPLC and the conversion of cellulose was monitored by size exclusion chromatography (SEC) and difference of weight. To our delight, after 3 h of irradiation, glucose was formed in 30% mass yield (Scheme 1). The reaction was fully selective to glucose, no other product was detected either by HPLC or mass spectrometry (Fig. S1†). ^1^H and ^13^C NMR confirmed the selectivity of the reaction and recorded NMR spectra of the crude product were rigorously similar to that of standard glucose (Fig. S2 and S3†). The absence of water-soluble low molecular weight oligosaccharides also suggests that the depolymerization occurs at the terminal position of the cellulosic chain. The yield of glucose also perfectly fits with the conversion of cellulose (30%), determined by measuring the difference of weight before and after HFUS treatment. Interestingly, the reaction also proceeded well at 40 °C and even 25 °C, without affecting the yield and the selectivity into glucose.

**Scheme 2:**
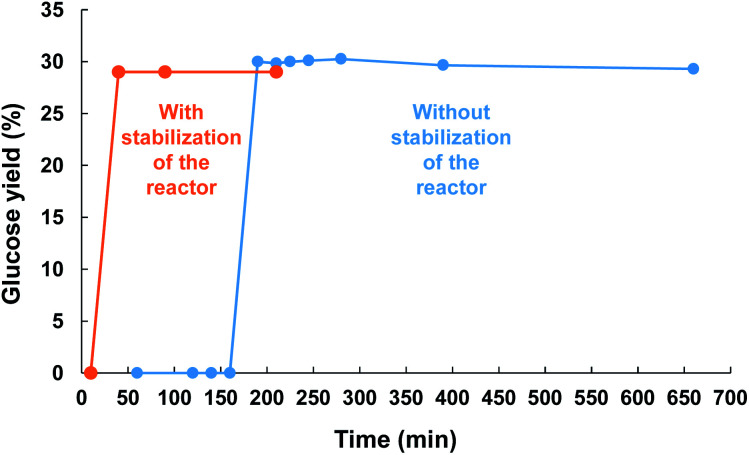
Kinetic profile of the reaction recorded at 60 °C under air (525 kHz, acoustic power: 0.36 W mL^−1^). Cellulose was introduced before (blue) or after (orange) warm-up of the reactor.

To rationalize the high selectivity into glucose, the remaining MCC was removed by filtration after 3 h and the as-obtained aqueous solution of glucose was subjected again to ultrasonic irradiations. Pleasingly, in the absence of cellulose particles, ultrasonic irradiation of the homogeneous solution of glucose led to quick degradation, through uncontrolled oxidation reactions, with gluconic acid being formed as a primary product, as observed earlier.^18^ This counter experiment is in agreement with the advanced hypothesis that, in the presence of MCC, radicals preferentially react with the cellulose particles rather than with released glucose in the bulk solution, thus optimizing the selectivity to glucose. Analysis of the recovered cellulose by SEC confirmed its depolymerisation with a reduction in the weight-average molecular weight (*M*_w_) and the number-average molecular weight (*M*_n_) from 49 × 10^3^ to 43 × 10^3^ g mol^−1^ and 38 × 10^3^ to 30 × 10^3^ g mol^−1^, respectively, after 3 h of reaction (Fig. S4†). Furthermore, analysis of the linking pattern of the recovered cellulosic material showed the exclusive occurrence of β-(1 → 4)-linked glucose units, as expected for cellulose. These results rule out the possible occurrence of repolymerization reactions, which would have led to the formation of 1,6 glycosidic linkages, as previously observed by the mechanocatalytic^12*g*^ or the NTAP technologies.^13*c*^ FT-IR and XPS analyses confirmed that no oxidation or C–C bond cleavage of the recovered cellulose occurred in our conditions, at least it is below the detection limit of our apparatus (Fig. S5 and S6†). In addition, XRD analysis did not show any change in the crystallinity index before and after HFUS, which strongly suggest that the *in situ* produced H˙ and ˙OH radicals selectively cleave the glycosidic bonds of cellulose (Fig. S7†).

The kinetic profile of the reaction was next monitored at 60 °C and revealed an induction period of about 3 h. At the moment, we have no rational explanation for this induction period. It seems it corresponds to the time for the HFUS reactor to reach its optimal efficiency (see ESI† for additional information). For instance, when ultrasonic irradiation of neat water was performed for 3 h prior to addition of cellulose, the induction period was reduced to only 10 min. After this period, cellulose was quasi instantaneously depolymerized to glucose, indicating that the depolymerisation rate of cellulose was very high. Subjecting cellulose to HFUS for a reaction time higher than 3 h did not result in a further improvement of the glucose yield, meaning that the depolymerisation of cellulose has stopped.

To get more insight on this phenomenon, the amount of cellulose suspended in water was varied from 0.5 wt% to 5 wt%. Independently of the cellulose loading, the depolymerisation reaction always stopped when the concentration of glucose reached 16.7 mmol L^−1^ (*i.e*. 0.3 g L^−1^), suggesting that released glucose inhibits the depolymerisation of cellulose induced by HFUS. We speculated that, at a certain concentration of glucose, this latter can trap the radicals on the cellulose particle surface. More information on this aspect is provided hereinafter. Complete depolymerisation of cellulose by HFUS should be theoretically feasible by switching from batch to continuous or semi-continuous processes. In this context, the cellulose recovered by filtration after the first batch was re-suspended in pure water and subjected again to HFUS. Interestingly, the depolymerisation occurred but stopped again at a concentration of glucose of 16.7 mmol L^−1^, leading to a cumulative yield of glucose of 60% after two runs (2 × 0.3 g of glucose, Scheme 1). These experiments could be reproduced four consecutive times leading to a quantitative depolymerisation of cellulose to glucose (Scheme 1).

**Scheme 3:**
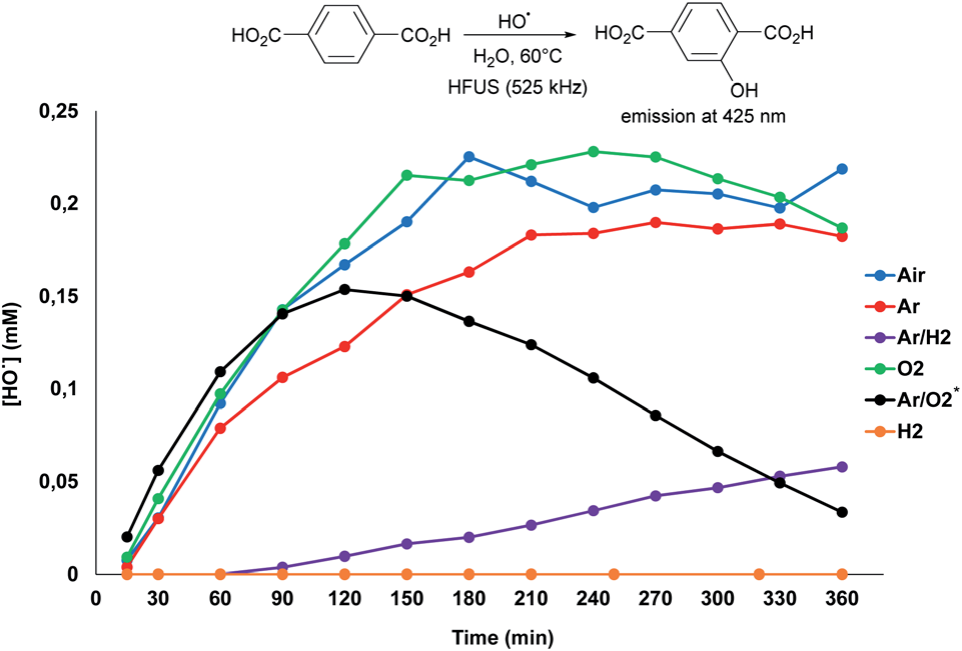
Titration of ˙OH radical using TPA (2 mM) as a function of the gaseous atmosphere. *The decrease in the ˙OH radical amount with time under Ar/O_2_ is due to the large production of ˙OH radicals which degrade TPA-OH used as a probe.

**Scheme 4:**
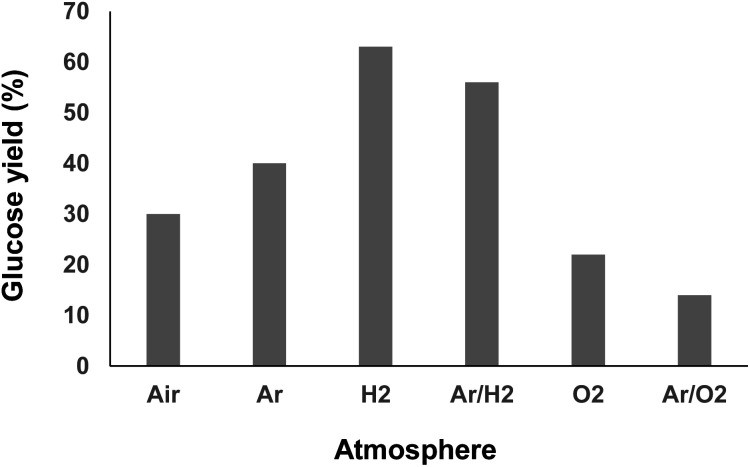
Maximum yield of glucose as a function of the gaseous atmosphere (60 °C, 525 kHz).

To explore the possibilities for further improving the glucose yield, we next investigated the reaction mechanism. It is widely accepted that irradiation of water at a high frequency leads to its homolytic dissociation to H˙ and ˙OH radicals.^15^ The H˙ radicals being difficult to observe due to fast recombination reactions, we first focused our investigations on the *in situ* produced ˙OH radicals. To this end, the formation of ˙OH radicals was monitored by fluorimetry using terephthalic acid (TPA) as an ˙OH radical scavenger (Scheme 3 and Fig. S8†).^19^ It is noteworthy that curves presented on Scheme 3 reach a maximum, and even decrease in some cases, which does not reflect the reality; it is actually due to the over-oxidation of the as-formed hydroxyterephthalic acid. Under air, the formation of ˙OH radicals was clearly evidenced by fluorimetry analysis. Ratio of specific heat, thermal conductivity and water solubility of gases impact the temperature of cavitation bubbles and thus the generation of radicals. In this context, the gaseous atmosphere was varied and changed from air to O_2_, Ar, H_2_ and mixtures of Ar/H_2_ and Ar/O_2_ (Scheme 3). In line with the state of the art on HFUS, the initial formation rate of ˙OH radicals was the highest in an Ar/O_2_ atmosphere.^20^ Conversely, addition of H_2_ to Ar dramatically inhibited the formation rate of ˙OH radicals, which was even inhibited when using neat H_2_ as a gaseous atmosphere, in line with previous report (Scheme 3).^21^ We noticed that the efficiency of HFUS-mediated cellulose depolymerisation was inversely proportional to the formation of ˙OH radicals, *i.e.* the higher the initial formation rate of ˙OH radicals, the lower the glucose yield (Scheme 4). For instance, cellulose was depolymerized to glucose with 63% yield under an H_2_ atmosphere (*vs.* 30% under air) while no ˙OH radical was formed in this case, suggesting that H˙ radicals are more likely to be involved in the reaction mechanism.

To independently assess the role of H˙ and ˙OH radicals in the reaction mechanism, control experiments were performed by adding two different radical scavengers during the ultrasonication of cellulose (Table 1). First, the ultrasonic irradiation of cellulose was performed under an Ar/H_2_ atmosphere in an aqueous solution of TPA (2.0 mM), with the aim of *in situ* trapping the ˙OH radicals. Consistent with our expectations, the glucose yield remained similar in the presence of 2.0 mM of TPA, supporting that ˙OH radicals have no major role in our case on the cellulose depolymerisation mechanism (Table 1, entry 2). This conclusion is also supported by FT-IR and XPS analyses which did not show oxidation, C–C bond cleavage or rearrangement of remaining cellulose. Next, the same reaction was performed by replacing TPA by carbon tetrachloride (CCl_4_). CCl_4_ is known to trap the H˙ radicals inside the cavitation bubbles.^16^ Despite the poor miscibility of CCl_4_ in water, addition of CCl_4_ completely inhibited the reaction, highlighting the important role of H˙ radicals in the depolymerisation of cellulose (Table 1, entry 3). This result is also consistent with our experiments under air, O_2_ and Ar/O_2_ for which the lowest yields in glucose were observed (Scheme 4). Indeed, H˙ radicals are known to be recombined with O_2_ to form ˙OH and ˙OOH radicals, as Niwano and Sivakumar previously observed by ESR spin trapping^21^ and dosimetry experiments,^19^ respectively. Hence, it is anticipated that the amount of free H˙ radicals is rather low under O_2_ and Ar/O_2_ atmosphere, explaining the low glucose yields obtained in these cases.

**Table 1:** Influence of H˙ and ˙OH radical scavengers in the HFUS-induced depolymerisation of cellulose

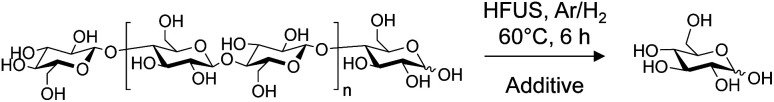		
Entry	Radical scavenger	Glucose yield (%)
1	—	52%
2	TPA	51%
3	CCl_4_	0

Altogether, these results show that the depolymerisation of cellulose observed under HFUS is enhanced under an H_2_ atmosphere, suggesting that H˙ radicals propelled onto the surface of cellulose particles can cleave the glycosidic bond of cellulose, presumably in a similar way as acid catalyst (protonation of the anomeric or the endocyclic oxygen of the terminal non-reducing glucopyranose unit).^22^ Depending on the nature of the gaseous atmosphere, the amount of H˙ radicals, and their recombination rate, can significantly differ. Although deeper investigations are needed at this stage to fully clarify the reaction mechanism, co-feeding the reactor with H_2_ seems to be an option to enhance the contact/reactivity of H˙ radicals with the cellulose surface and to ensure its extensive depolymerisation.

As above mentioned, at a certain concentration, glucose may interact with cellulose particle surfaces where it could locally trap radicals. Under oxygen free conditions, no change in pH (∼6.5) was observed, even at extended reaction times, ruling out a possible oxidation of glucose in this case. This was further supported by ^13^C NMR and mass spectrometry investigations, which did not show the formation of any C=O group. Furthermore, unlike TPA, when 16 mM of glucose was initially added to cellulose, the depolymerisation was completely inhibited, suggesting that glucose similarly behaves as CCl_4_ and scavenge H˙ radicals on the cellulose surface. As an evidence to it, we observed that, at extended ultrasonic irradiation time under H_2_ or Ar/H_2_ atmospheres, the glucose yield gradually decreased and fructose was concomitantly formed (Fig. S9†). This observation was supported by HPLC analysis and by ^1^H/^13^C NMR investigations, which clearly evidenced the selective formation of glucose and fructose in a 83/17 (glucose/fructose) ratio (Fig. S9–S11†). Although additional experiments are needed to fully rationalize the reaction mechanism, the absence of pH change and the partial isomerization of glucose to fructose under oxygen free atmosphere are strong arguments in favour of the involvement of H˙ radicals. This claim is also in agreement with our previous report which show, by density functional theory calculations, that H˙ radicals promote the ring opening of glucose,^17*a*^ a known key step in its isomerization to fructose.

Interestingly, previously reported technologies involving radical activation of cellulose such as photolysis, UV excitation, radiolysis by X-ray, γ-ray irradiation, electron beam, *etc.*,^23^ led also to glycosidic bond cleavage but with uncontrolled side dehydration, recombination or rearrangement of the glucosyl unit. Hence, *in situ* produced radicals alone cannot explain the very high selectivity into glucose observed using HFUS. One may suspect that, as in the case of supercritical water or the mechanocatalytic process, the implosion of cavitation bubbles on the surface of cellulose locally provides enough physical forces (pressure, shock waves, *etc.*) capable of inducing a conformational change of the glycosidic bond,^7*a*^ which then become much more reactive.

## Conclusions

We show here that HFUS is an alternative technology capable of selectively depolymerizing cellulose to glucose. The possible transfer of radicals produced inside the cavitation bubbles onto the cellulose particle surfaces was an efficient mean to prevent the concomitant side-degradation of glucose into the bulk solution. In contrast to previously reported technologies, this work is distinguished in that (i) no catalyst was needed, (ii) no external source of heating was required, and (iii) it proceeds in a very selective fashion. A deeper understanding of the reaction mechanism suggest that H˙ radicals are more likely to be responsible for the depolymerisation of cellulose. By recirculating unreacted cellulose into the HFUS reactor, it was possible to selectively and quantitatively depolymerize cellulose to glucose, which constitute one of the rare cases reported so far. A combined experimental-theoretical approach is now the topic of current investigations in our groups in order to get more insight on the reaction mechanism, including the observed induction period in Scheme 2.

## Conflicts of interest

There are no conflicts to declare.

## Supplementary Material

SC-011-D0SC00020E-s001
