# Supplementary material for: Selective radical depolymerization of cellulose to glucose induced by high frequency ultrasound
Source: Chem Sci. 2020 Feb 6;11(10):2664–9. doi: 10.1039/d0sc00020e (PMC8157487; doi:10.1039/d0sc00020e)
Supplement: SC-011-D0SC00020E-s001 [file SC-011-D0SC00020E-s001.pdf]

Electronic Supplementary Material (ESI) for Chemical Science.
This journal is © The Royal Society of Chemistry 2020

SUPPORTING INFORMATION

Selective radical depolymerization of cellulose to glucose induced by high frequency ultrasound

Somia Haouache,[a,d] Ayman Karam,[a] Tony Chave,[b] Jonathan Clarhaut,[a] Prince N. Amaniampong,[a] José M. Garcia Fernandez,[c] Karine De Oliveira Vigier,[a] Isabelle Capron[d] and François Jérôme*[a]

[a.] *Institut de Chimie des Milieux et Matériaux de Poitiers, Université de Poitiers-CNRS, 1 rue Marcel Doré, 86073 Poitiers (France). E-mail: francois.jerome@univ-poitiers.fr.*

[b.] *Institut de Chimie Séparative de Marcoul, 30207 Bagnols-sur-Cèze (France)*

[c.] *Institute for Chemical Research, CSIC, University of Sevilla, Americo Vespucio 49, Isla de la Cartuja, 41092 Sevilla (Spain).*

[d.] *INRA, Site de la Géraudière, 44316 Nantes (France)*

Experimental procedure……… S2
High Performance Liquid chromatography ((HPLC)……… S4
Matrix-assisted laser desorption/ionization-time-of-flight (MALDI-TOF)……… S5
Nuclear magnetic resonance (NMR)……… S7
Cellulose methylation analysis……… S8
Size Exclusion Chromatography coupled to Multi-Angle Laser Light Scattering and Refractive Index (SEC-MALLS-RI) detection……… S9
FT-IR characterization……… S10
X-ray photoelectron spectroscopy (XPS)……… S11
X-ray diffraction (XRD)……… S12
Fluorimetry……… S13
HPLC and ^{13}C NMR under Ar/H_2 atmosphere……… S14
References……… S15

Experimental procedure

Microcrystalline cellulose (1g) was suspended in 100 mL of water subjected to an ultrasonic irradiation at 525 kHz (amplitude: 100%) in a high-frequency ultrasonic reactor from SinapTec Ultrasonic Technology (NextGen Lab 1000, standby power $P_0 = 13.9$ W, nominal electric power of the generator $P_{elec} = 46.1$ W, acoustic power in water of $P_{acous.vol} = 0.36$ W.mL^{-1}) (Picture S1). To avoid sedimentation of cellulose particles, ultrasonication of water was always started before addition of cellulose. During the sonication, the resulting mixture was kept at 60°C and gently stirred with a magnetic stirring bar at 150 rpm.

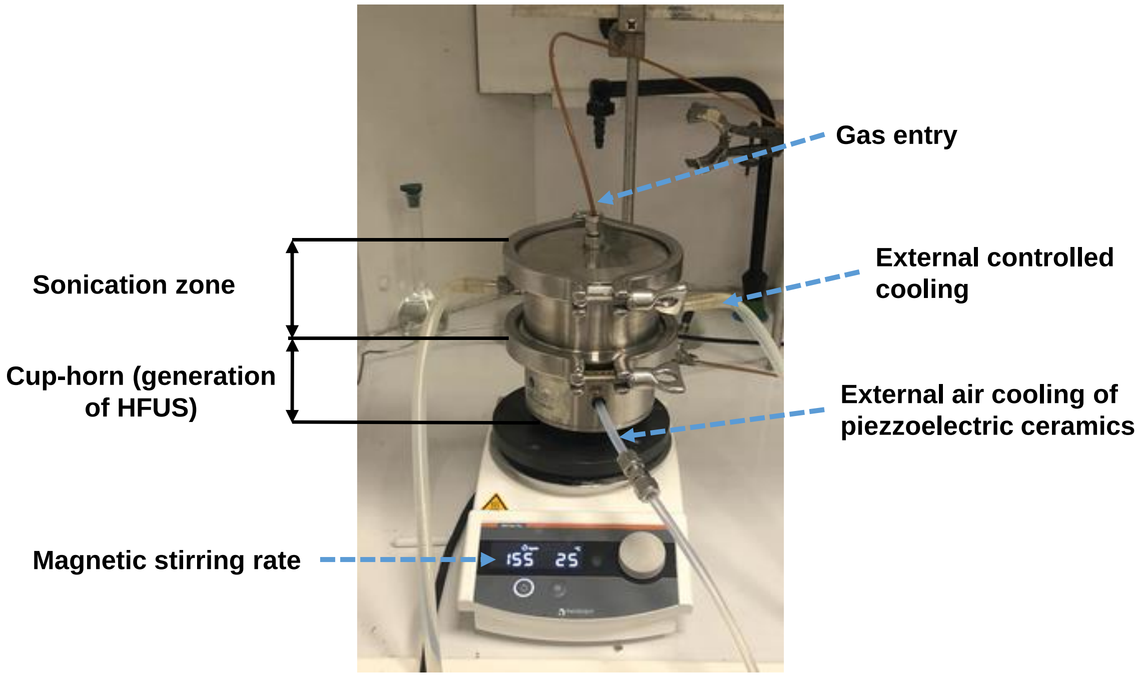

Picture S1. Photography of the HFUS used in this work

Experiments were carried out under atmospheric pressure by bubbling air, oxygen, argon, hydrogen or mixtures of argon/hydrogen (70/30) and argon/oxygen (80/20). At the end of the reaction, the unreacted cellulose was removed by centrifugation and the aqueous solution of glucose was analyzed by HPLC, mass spectrometry and NMR. For NMR investigations, the aqueous solution of glucose was first freeze-dried, yielding glucose as a white powder, and then re-dissolved at room temperature in D_2O.

Note: as mentioned in the article text, an induction of period of about 3 h was observed in the kinetic profile of the reaction (Scheme 1). This induction period is not due to cellulose but to the reactor itself. We did extra analysis with the aim of getting more insights on this aspect. As it is difficult to monitor the formation of radical H•, we monitored the formation of H_2O_2,

resulting from the recombination of •OH radicals, as a function of the ultrasonic irradiation time (without cellulose). We observed in the graph S1b, a linear and constant increase of H_2O_2, indicating that the homolytic dissociation of water in the cavitation bubbles takes place as soon as the ultrasound starts (no induction period). This result is in line with Scheme 3 on dosimetry experiments. Note that we also performed a reaction by initially co-adding H_2O_2 with cellulose, but it had no impact on the induction period, ruling out a possible reaction of cellulose with accumulated H_2O_2 inside the reactor. However, we noticed that the induction period varies according to the nature of the gas bubbled. For instance, when air was replaced by Ar/H_2 or H_2 the induction period was decreased from 3 h to less than 1 h. Under bubbling of O_2, this induction period is 2 h. Hence, we suspect that this induction period may correspond to problems of mass transfer (*i.e.* dissolution and diffusion of the gas into water), homogenization of the cavitation bubble cloud, etc. However, these are only hypotheses and, so far, our results do not let us to rationalize this induction period and we are currently working on this aspect with expert of ultrasound (T. Chave's group in the author list), mainly because we observe the same trend on other HFUS-mediated reactions we are currently investigating in the lab.

Characterization of the HFUS by titration of H_2O_2 without cellulose

H_2O_2 was titrated by UV-visible spectroscopy (Thermo Fisher Evolution 60S) using $TiOSO_4$. This latter reacts with H_2O_2 to form a yellow-colored Ti(IV)-H_2O_2 complex with a typical adsorption at 412 nm.

$TiO^{2+} + H_2O_2 \rightarrow TiO(O_2) + 2H^+$

In a typical procedure, 553 mg of $TiOSO_4$ was dissolved in 2.8 mL of H_2SO_4 (96%) and water was added at 50°C up to a total volume of 100 mL was reached.

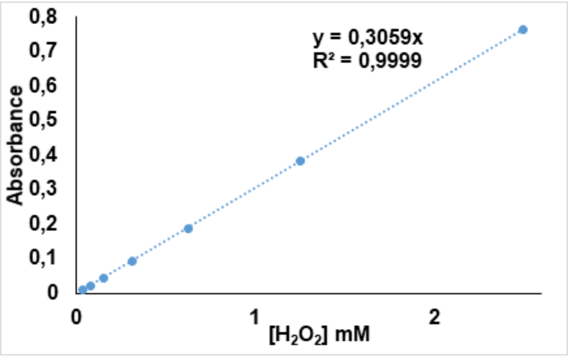

Graph S1a : calibration curve

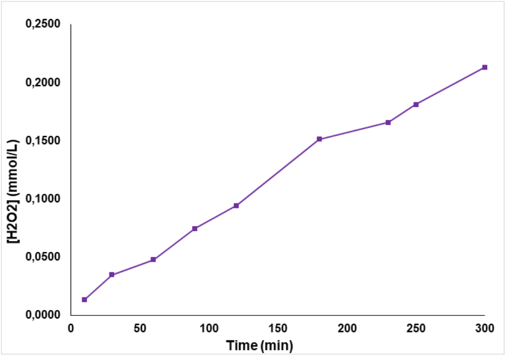

Graph S1b : titration of H_2O_2 without cellulose *vs* time (525 kHz, amplitude 100%, 60°C)

High Performance Liquid chromatography (HPLC)

The reaction media was analyzed by a Shimadzu HPLC equipped with a ZORBAX NH2 apolar type column, a pump (LC-20AT), a thermostated autosampler (SIL-10A) and an oven heated at 40 °C (CTO-20AC). The eluted compound were detected and quantified using a refractive index detector (RID-10A). The mobile phase consists of water and acetonitrile (20:80) injected at a flow rate of 0.8 $mL.min^{-1}$. Standard solutions (glucose, fructose) were prepared from commercial products purchased from Sigma-Aldrich. The retention times, mathematic expression of the calibration curve and R^2 are summarized in Table S1.

Table S1: Retention times, mathematic expression of the calibration curve and R^2 of fructose and glucose.

Compound	Retention time (min)	Mathematic expression	R^2
Glucose	16.7	y = 4035.6x	0.9992
Fructose	13.3	y = 16197x	0.9984

y = peak area and x = hexose concentration

Matrix-assisted laser desorption/ionization-time-of-flight (MALDI-TOF)

The unreacted cellulose remaining after ultrasonic irradiation was removed by centrifugation. The as-obtained solution was freeze-dried and then analysed by MALDI-TOF. An ionic preparation comprised of 2,5-dihydroxybenzoic acid (DHB) and *N*,*N*-dimethylaniline (DMA) was used as the MALDI matrix, as described by Ropartz et al [1]. Typically, the matrix consists of a solution of DHB at 100 mg.ml^{-1} prepared in H_2O/acetonitrile/ DMA (1:1:0.02). The samples (1 μL) were deposited and then covered by the matrix (1 μL) on a polished steel MALDI target plate. MALDI measurements were then performed on a rapifleX MALDI- TOF spectrometer (Bruker Daltonics, Bremen, Germany) equipped with a Smartbeam 3D laser (355 nm, 10000 Hz) and controlled using the Flex Control 4.0 software package. The mass spectrometer was operated with positive polarity in reflectron mode. Spectra were acquired in the range of 180–2500 m/z (Figure S1a).

Zooming around the expected masses, we observe, a peak corresponding to a sodium adduct of glucose (*m/z* = 203, Figure S1b) and no other peak that could correspond to glucose oxidation or oligosaccharides (dimer and trimer) were observed, as shown on the zooms around DP2 (Figure S1c), DP3 (Figure S1d) and in the *m/z* = 1000-2500 range (Figure S1e). Peaks at *m/z* = 253, 275, 313, 361, 372, 416, 501, 545, 708, 721, 891 and 955 corresponding to clusters from the MALDI matrix (DHB).

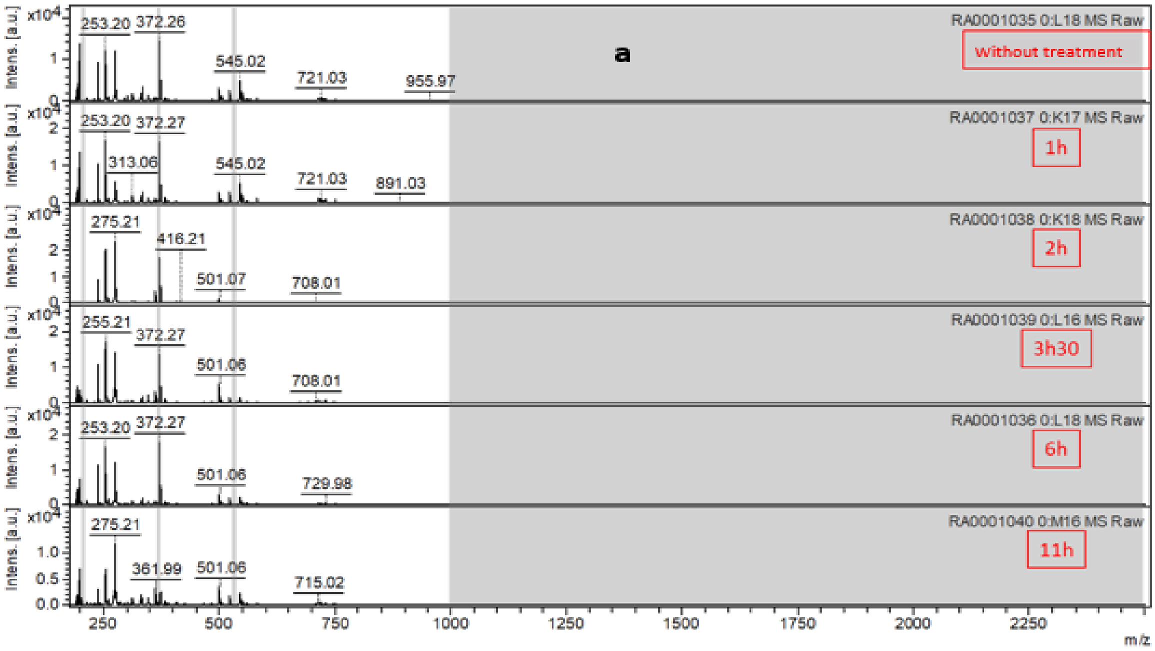

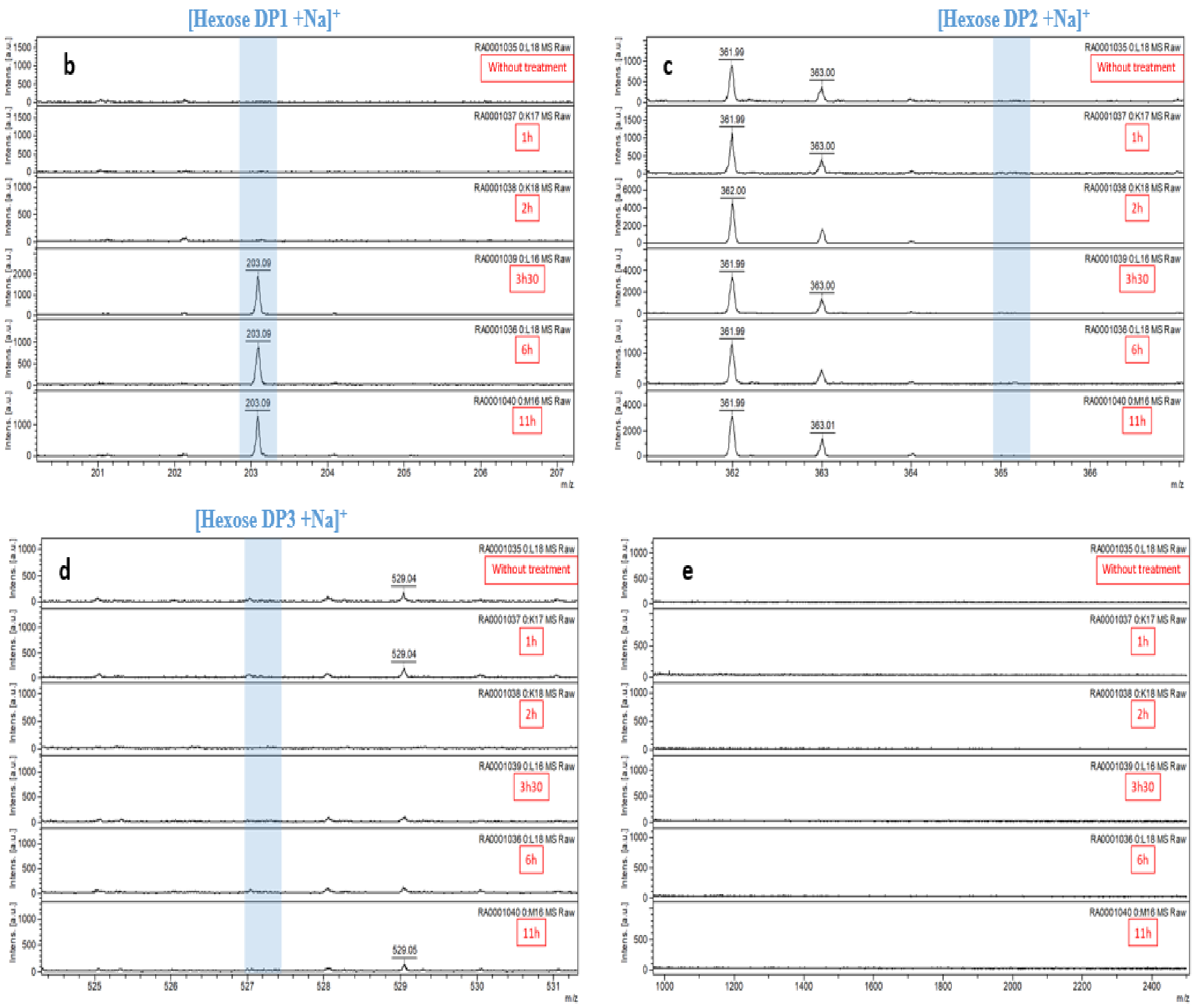

Figure S1: Comparison of MALDI-TOF spectra, m/z range 180-2500 (a); 200-207 (b); 361-367 (c); 524-531 (d); 1000-2500 (e) of the solution obtained from MCC without ultrasonic treatment and after 1h, 2h, 3h30, 6h and 11h of ultrasonic irradiation under air.

Nuclear magnetic resonance (NMR)

NMR analyzes (^{1}H, ^{13}C) were recorded on a Bruker Ultrashield 500 plus device (500 MHz). For this analysis, the residual MCC after ultrasonic irradiation was removed by centrifugation. The as-obtained solution was then freeze-dried and solubilized in D_2O.

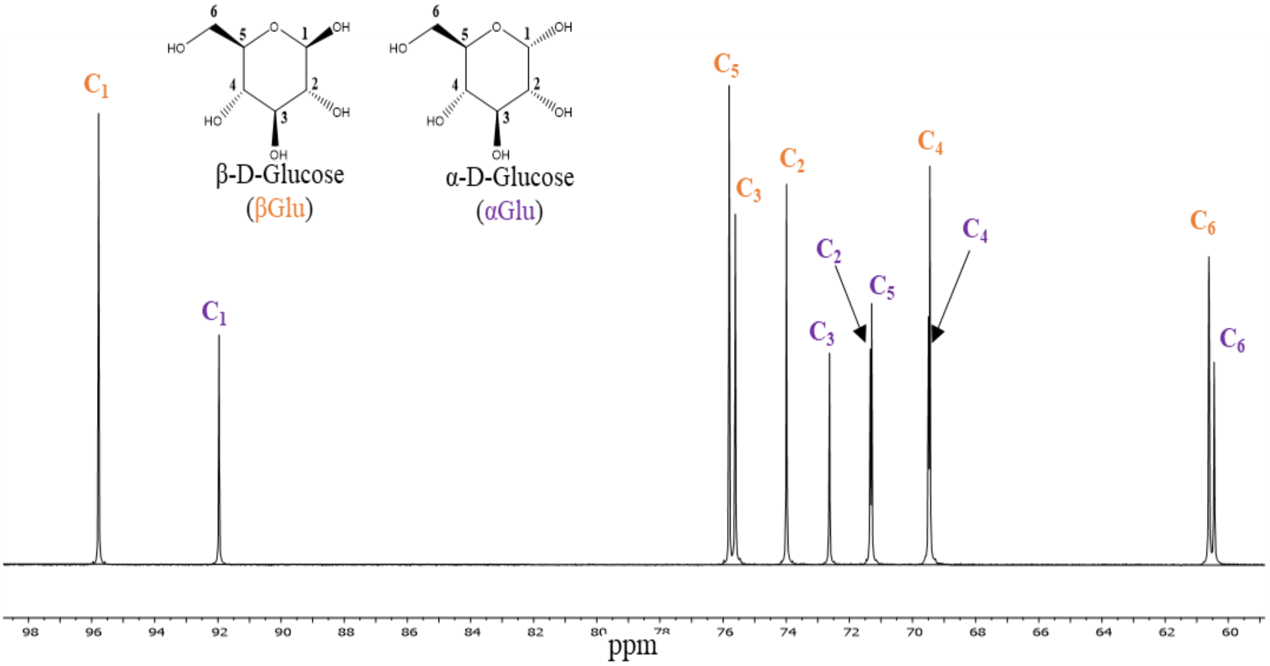

Figure S2: ^{13}C NMR spectrum of the crude solution obtained after ultrasonic irradiation (525 kHz) of cellulose for 3h at 60°C under Air

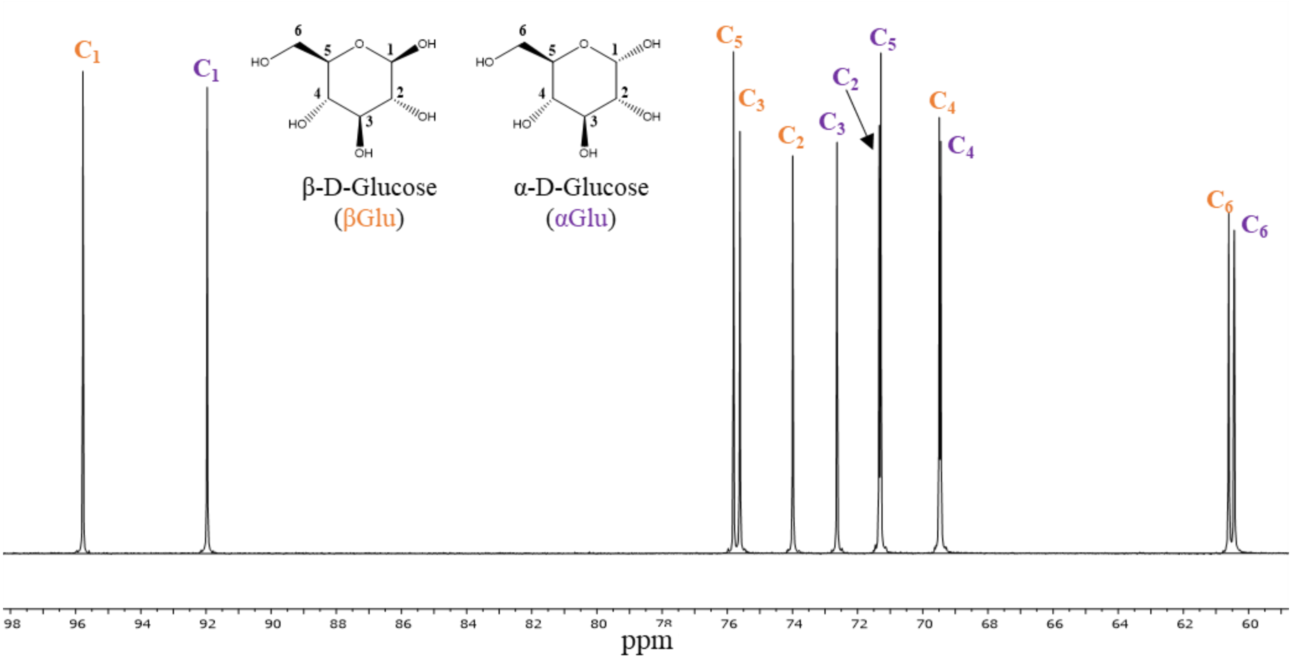

Figure S3: ^{13}C NMR spectrum of commercial D-glucose in D_2O. Assignment was done on the basis of a previous work reported on ref [2]

Cellulose methylation analysis

The cellulose samples after HFUS were permethylated and analyzed for linkage type by the alditol acetate method of Harris *et al* [3]. Briefly, the samples were dried under vacuum over phosphorus pentaoxide for 16 h. Me_2SO was added under Ar to the dried, finally grounded sample (1-5 mg), which was next subjected to two rapid, preliminary methylations by sequential addition of potassium methylsulphinyl carbanion (20 μL), ice-cold methyl iodide (15, μL), potassium methylsulphinyl carbanion (60 μL) and ice-cold methyl iodide (15, μL), with vigorous mixing in between. The dissolution thus obtained was subjected to a final methylation reaction by treatment with methylsulphinyl carbanion (200μ). After 10 min, the solution is cooled to 0 ºC and ice-cold methyl iodide (150 μL) was added. The mixture was allowed to warm to room temperature over 10 min. A 2: 1 (v/v) chloroform-methanol mixture (3 mL) and water (2 mL) was then added and the mixture was vigorously stirred and centrifuged (200 g, 30 s) to aid phase separation. The upper phase was separated by aspiration and the washing procedure was repeated 4 times using water (2 mL). 2,2-Dimethoxypropane (2 mL) and 18 M acetic acid (20 μL) were added and the mixture was placed in a water bath (90 ºC) and let evaporate to a volume of ≈200 μL. The remaining solvent was evaporated under a stream of argon. The resulting crude permethylated product was next subjected to a deuteroboration (NaBD4)–acetylation (Ac_2O/TFA) reaction sequence prior to GC-MS analysis. This protocol affords the corresponding sugar alditols, labelled with deuterium at C-1, methylated at non-glycosylated positions and bearing acetyl groups at positions that were originally glycosylated in the starting oligomer, which can be unequivocally assigned from the corresponding fragmentation patterns in MS by comparison with authentic standards. In all the analyzed samples, only the peak corresponding to 1,4,-di-*O*-acetyl-2,3,6-tri-*O*-methylsorbitol was detected in the corresponding chromatograms, indicating that exclusively 1→4 glycosidic linkages occurred in the samples. This is in agreement with the linear structure of cellulose and discards that reversion reaction takes place under the HFUS depolymerization conditions.

Size Exclusion Chromatography coupled to Multi-Angle Laser Light Scattering and Refractive Index (SEC-MALLS-RI) detection

The determination of molar mass distribution of chains of cellulose constituting the samples was carried out at room temperature using OMNISEC SYSTEM Malvern. The size exclusion chromatography (SEC) (OMNISEC Resolve, Malvern) system was coupled to a multi-angle laser light scattering 20 (MALLS) (Malvern) and OMNISEC Reveal devices (Malvern). The SEC columns used were Viscoteck Tguard, LT4000L, LT5000L and LT7000L. The mobile phase was *N*,*N*-dimethylacetamide (DMAc) (HPLC grade) containing lithium chloride (LiCl) (0.9 %w/v). Before use, the eluent was filtered through 0.6µm polypropylene prefilters. This eluent was selected because it solubilizes cellulose without significant depolymerization during the dissolution process or during storage at room temperature for long periods.[4][5] Calculation of weight and number average molar masses ($\overline{M}_w$,$\overline{M}_n$) and polydispersity ($\overline{M}_w/\overline{M}_n$) of samples were performed with a dn/dc value of 0.136 mL/g and determined with OMNISEC software (v. 10.30) with Zimm extrapolation of order 2 (**Figure S4**).

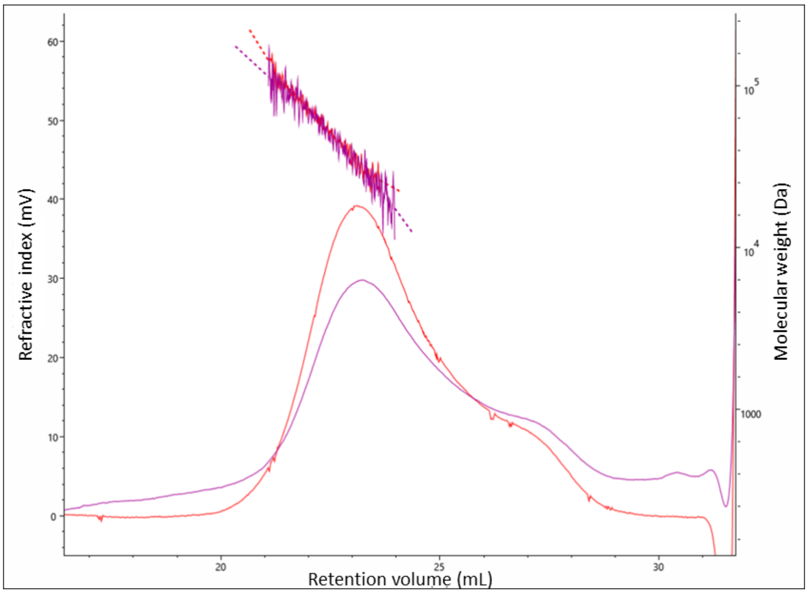

Figure S4: Dissolution profiles of MCC before (red) and after (purple) ultrasonic irradiation under air obtained by SEC-MALLS-DRI.

FT-IR characterization

FT-IR analysis was carried out using a Perkin Elmer Spectrum One FT-IR Spectrometer infrared spectrometer coupled with an ATR module (Perkin Elmer Universal ATR sampling accessory). The scans were recorded between 4000 cm^{-1} and 650 cm^{-1} (**Figure S5**).

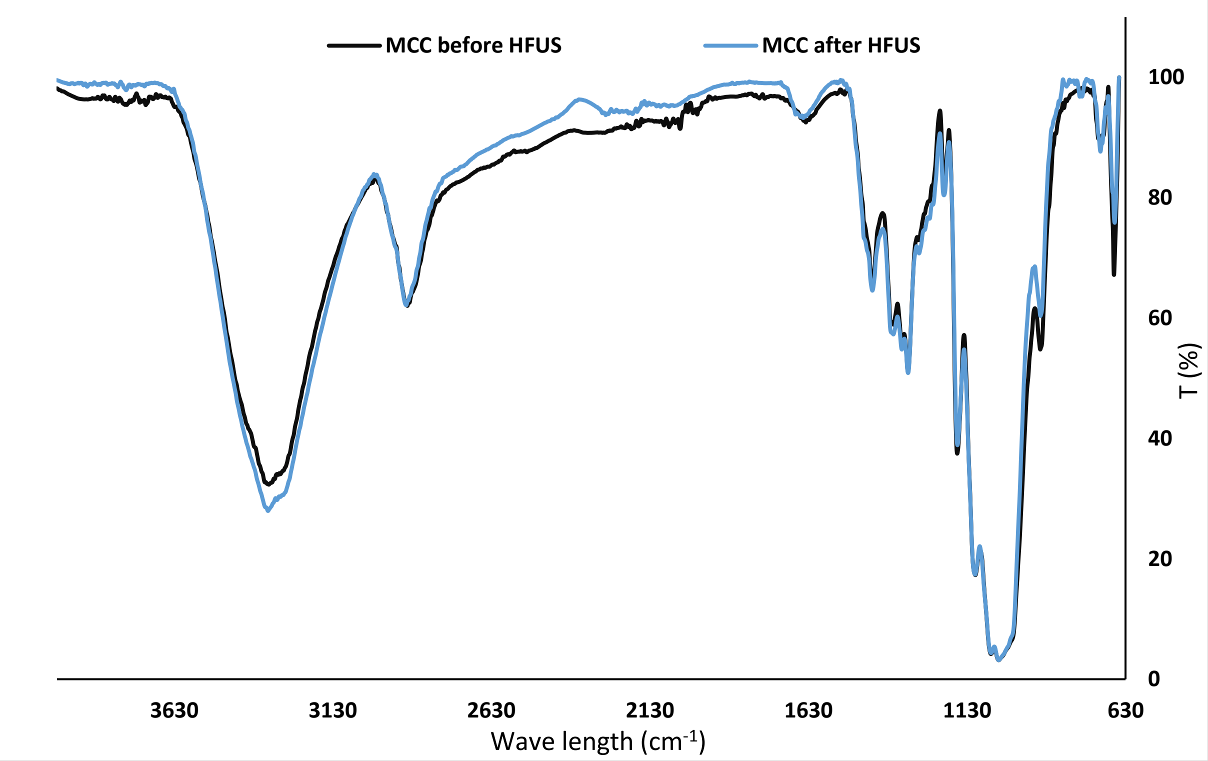

Figure S5: FT-IR spectrum of MCC before and after ultrasonic irradiation under air (HFUS = High Frequency Ultrasound)

X-ray photoelectron spectroscopy (XPS)

X-ray photoelectron spectroscopy (XPS) of cellulose before and after ultrasonic irradiation was performed on a Kratos Axis Ultra DLD apparatus equipped with a hemispherical analyzer and a delay line detector. The spectra were recorded using an Al monochromated X-ray source (10 kV, 15 mA) with a pass energy of 40 eV (0.1 eV per step) for high resolution spectra, and a pass energy of 160 eV (1 eV per step) for survey spectrum in hybrid mode and slot lens mode, respectively. XPS spectra were calibrated with respect to the C 1s orbital at 284.8 eV.

According to the XPS results, no surface oxidation of cellulose after ultrasonic irradiation was observed.

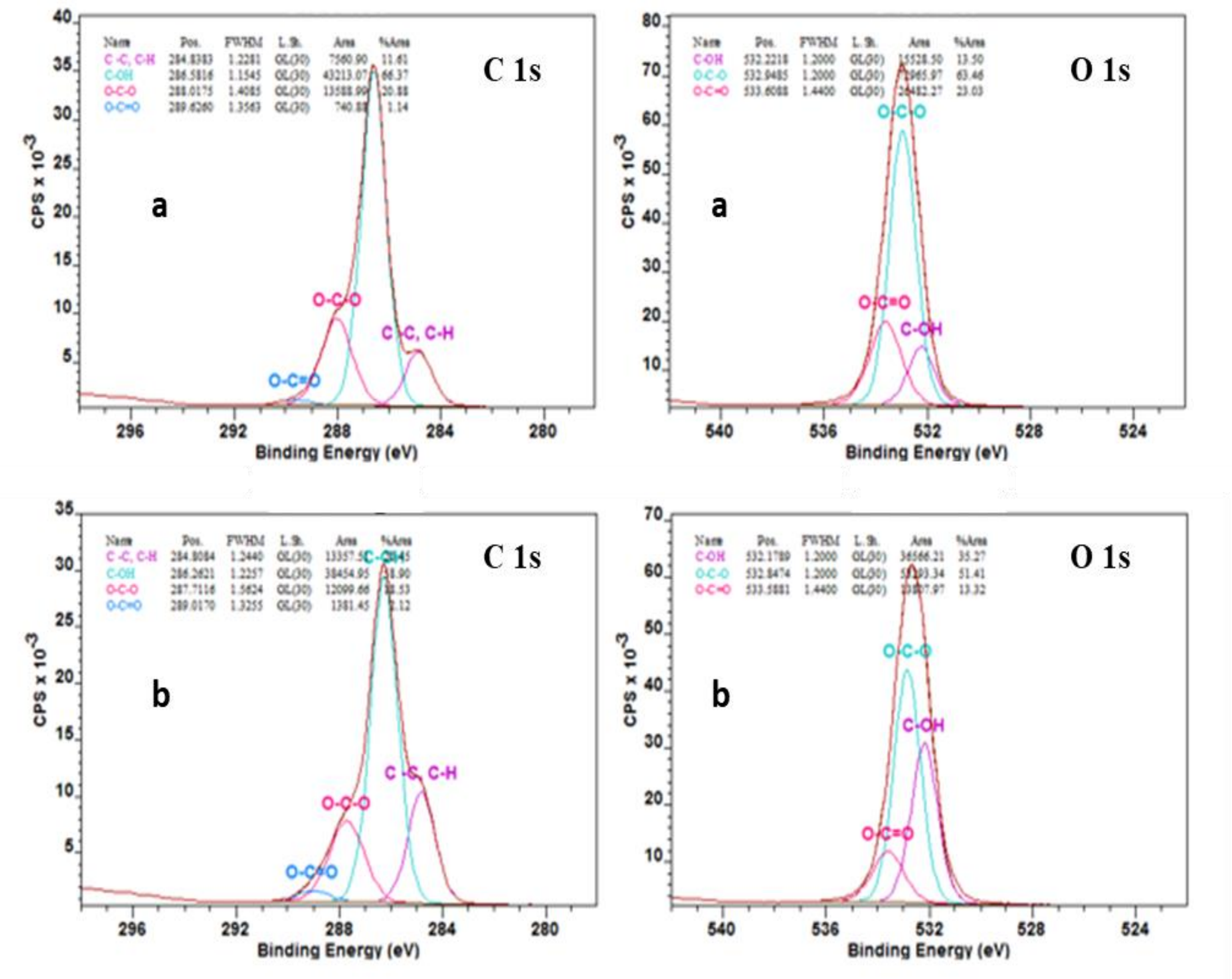

Figure S6: XPS spectra of cellulose before (a) and after (b) ultrasonic irradiation under air

X-ray diffraction (XRD)

The X-ray diffractometer used is an "EMPYREAN" (PANalytical) equipped with a copper tube (characteristic wavelength: λ ($K\alpha_1$) = 0.1540562 nm), a "fast" linear detector, called "X'Celerator", and a platinum (or "spinner") allowing a rotation of the sample. The measurement are made between 5° and 50° in 2Thétas, and the displacement was fixed at 0.1° for an accumulation of 600 s per step.

The crystallinity index (I_{CR}) of the samples was calculated as in Langford et Wilson [6] from the XRD spectra using the following equation

$$I_{CR} = \frac{I_{200} - I_{AM}}{I_{200}} \times 100$$

Where I_{200} is the intensity of the crystal peak located at 22.6° corresponding to the plane (200) and I_{AM} is the intensity of the valley situated between the two peaks located at 22.6° and 15.5° corresponding to the amorphous intensity.

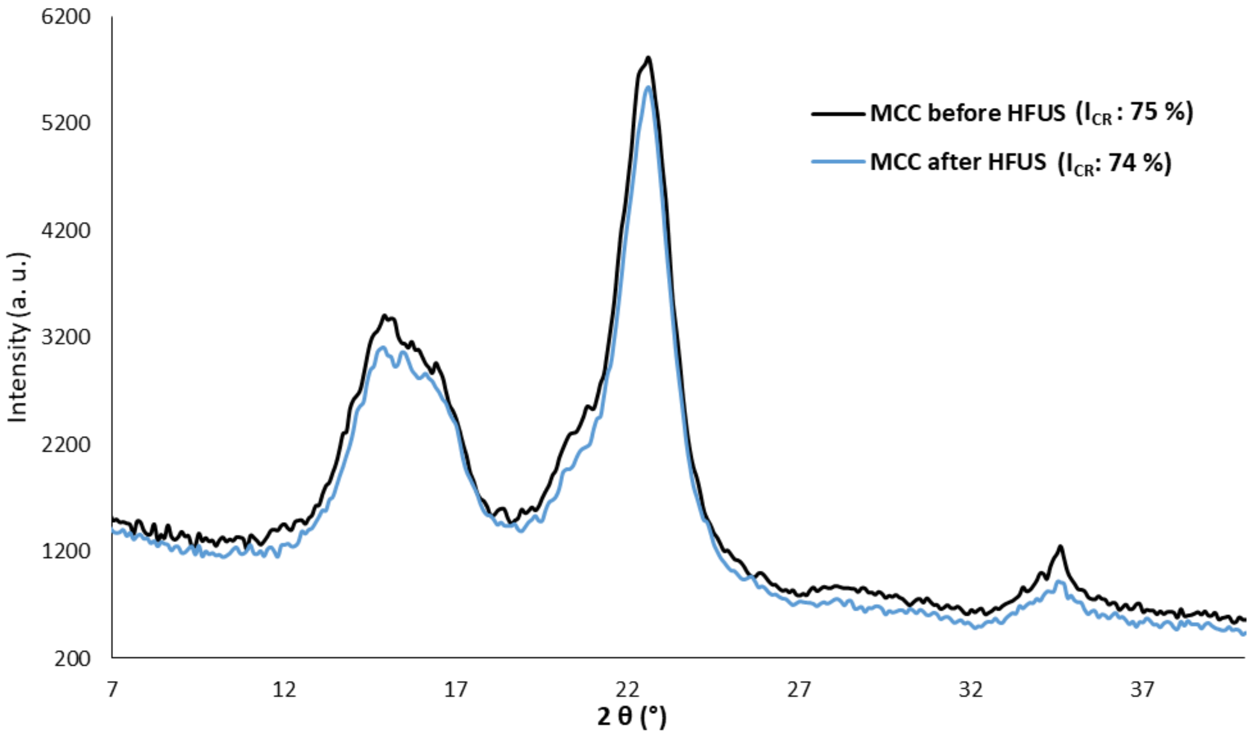

Figure S7: XRD patterns of MCC before and after ultrasonic irradiation under air (HFUS = High Frequency Ultrasound)

Fluorimetry

The amount of OH radical formed *in situ* under the different gases was determined by fluorimetry using terephthalic acid (TPA) as scavenger of OH radicals [7] (Figure S8). The fluorescences (λ_{ex}: 315 nm, λ_{em}: 425 nm) were determined on a FlexStation 3 (Molecular Devices) multi-mode 96-well microplate reader. The calibration was performed with a solution of 2-hydroxyterephthalic acid (2HTPA).

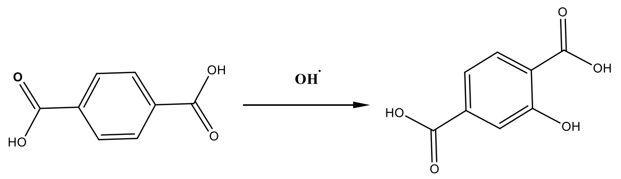

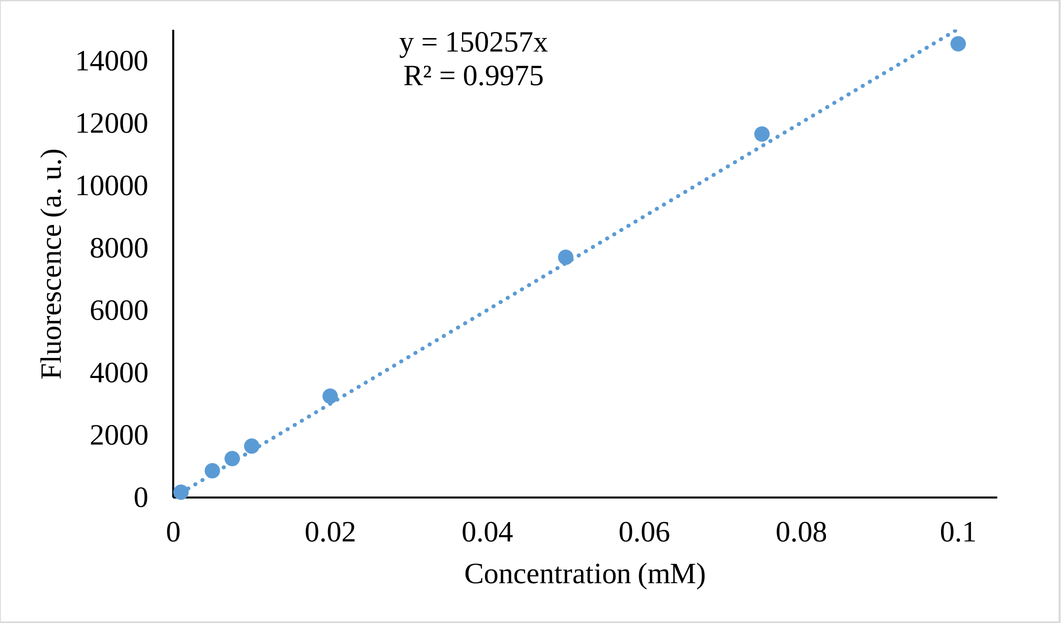

Figure S8: Calibration with 2-hydroxylterephthalic acid

HPLC and ^{13}C NMR under Ar/H_2 atmosphere

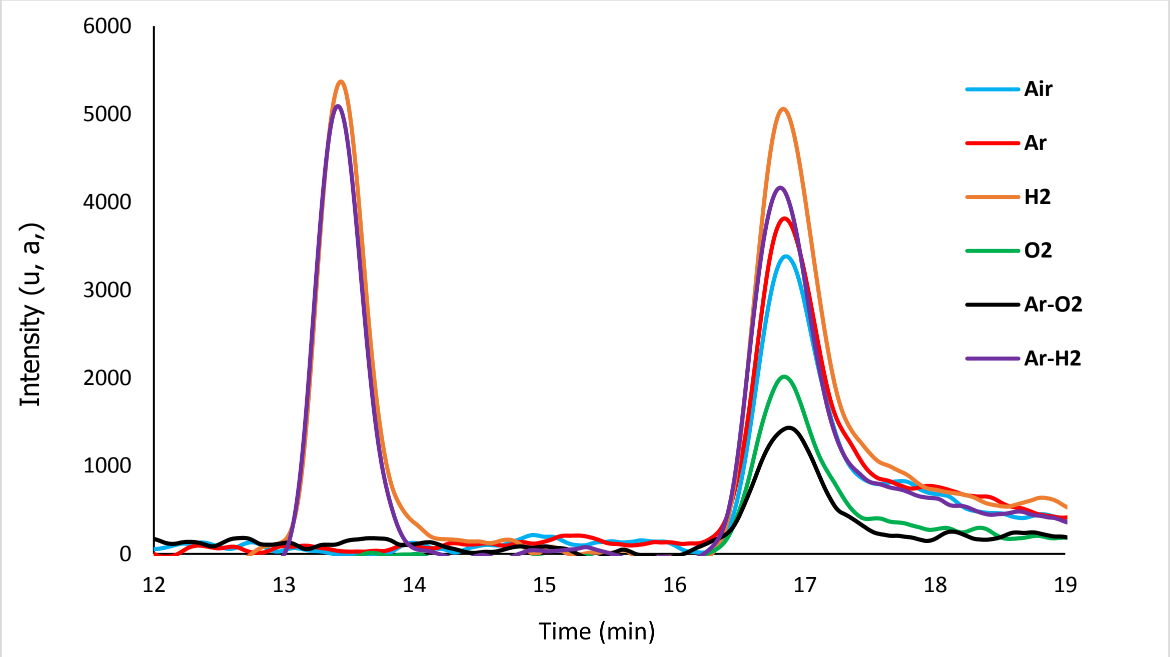

Figure S9: HPLC chromatogram for the products of the reaction (fructose at 13.3 min) and (glucose at 16.7 min) under different gases

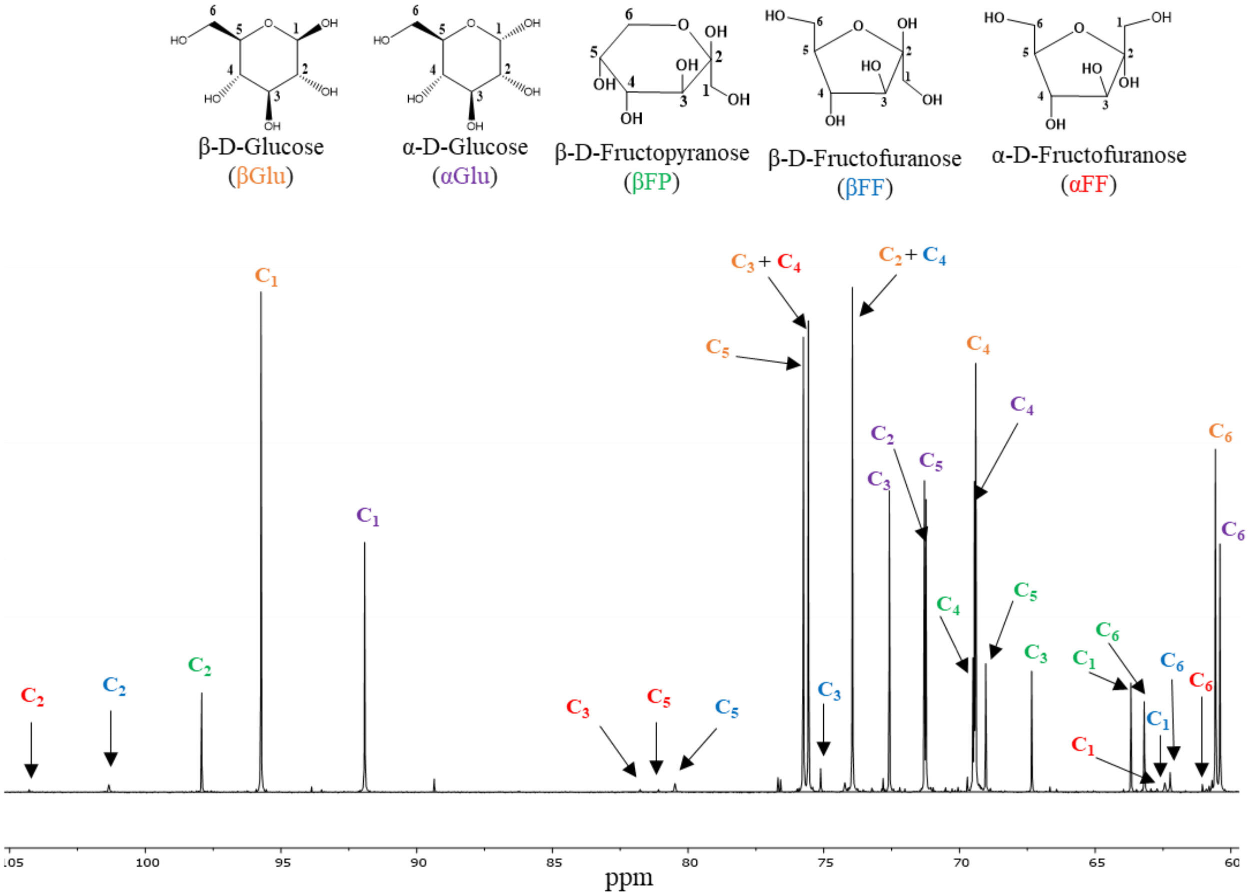

Figure S10: ^{13}C NMR spectrum of the crude solution obtained after 6 h of ultrasonic irradiation of cellulose under Ar/H_2

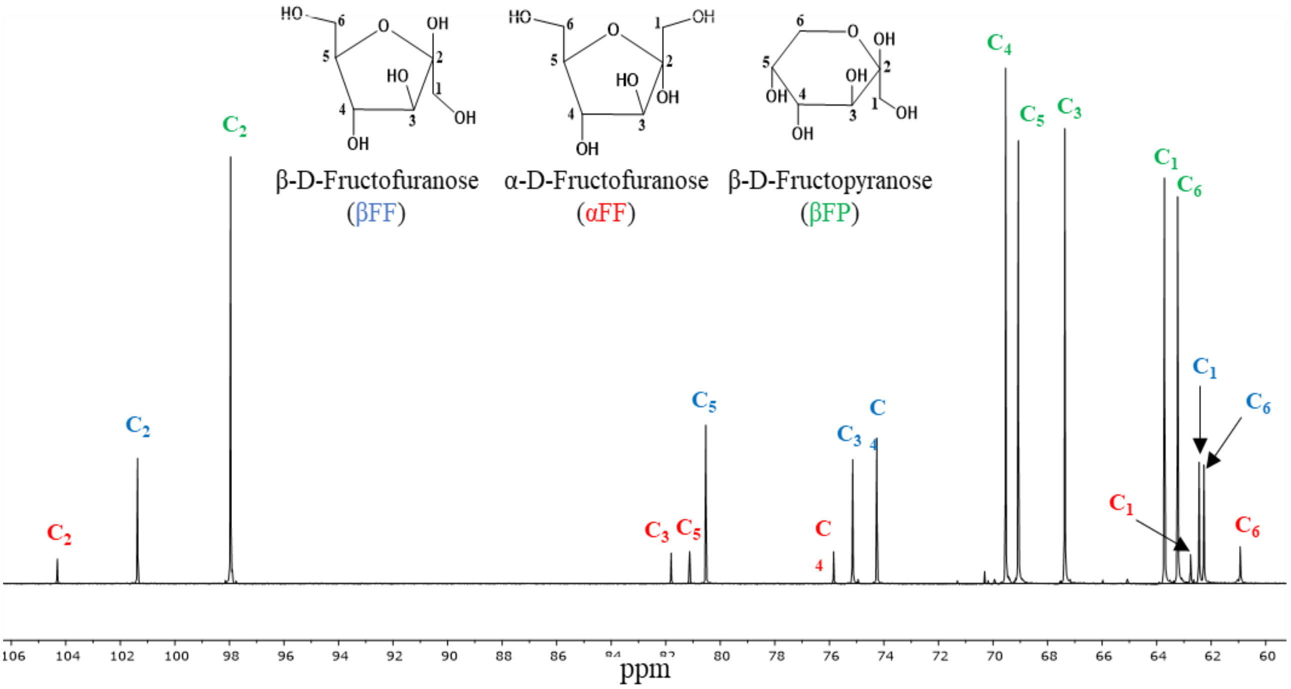

Figure S11: ^{13}C NMR spectrum of commercial D-fructose in D_2O. Assignment was done on the basis of a previous work reported on ref [8]

References

[1] D. Ropartz *et al.*, « Performance evaluation on a wide set of matrix-assisted laser desorption ionization matrices for the detection of oligosaccharides in a high-throughput mass spectrometric screening of carbohydrate depolymerizing enzymes », *Rapid Commun. Mass Spectrom.*, vol. 25, nº 14, p. 2059–2070, 2011.

[2] http://www.hmdb.ca/spectra/nmr_one_d/1152

[3] J. H. Harris, R. Henry, A. E. Blakeney, B. A. Stone, Carbohydr. Res. 1984, 127, 59-73.

[4] A.-L. Dupont et G. Harrison, « Conformation and dn/dc determination of cellulose in N, N-dimethylacetamide containing lithium chloride », *Carbohydr. Polym.*, vol. 58, nº 3, p. 233–243, 2004.

[5] M. Yanagisawa et A. Isogai, « SEC- MALS- QELS study on the molecular conformation of cellulose in LiCl/amide solutions », *Biomacromolecules*, vol. 6, nº 3, p. 1258–1265, 2005.

[6] J. I. Langford et A. J. C. Wilson, « Scherrer after sixty years: a survey and some new results in the determination of crystallite size », *J. Appl. Crystallogr.*, vol. 11, nº 2, p. 102–113, 1978.

[7] Y. Iida, K. Yasui, T. Tuziuti, et M. Sivakumar, « Sonochemistry and its dosimetry », *Microchem. J.*, vol. 80, nº 2, p. 159–164, 2005.

[8] J. C. Edwards, J. M. Hunter, et B. V. Nemzer, « Multinuclear NMR of calcium fructoborate complex—structure, stability, and quantitation in the presence of other ingredients, excipients or adulterants », *J Food Res*, vol. 3, nº 3, p. 115–131, 2014.
